# Advances in High-Field BOLD fMRI

**DOI:** 10.3390/ma4111941

**Published:** 2011-11-02

**Authors:** Markus Barth, Benedikt A. Poser

**Affiliations:** 1 Radboud University Nijmegen, Donders Institute for Brain, Cognition and Behaviour, Nijmegen 6525 HP, The Netherlands; E-Mail: poser@hawaii.edu; 2 Erwin L. Hahn Institute for Magnetic Resonance Imaging, University Duisburg-Essen, Essen 45141, Germany; 3 Department of Medicine, John A. Burns School of Medicine, University of Hawaii, Honolulu, HI 96785, USA

**Keywords:** BOLD fMRI, high field, 7 Tesla, BOLD response, 2D EPI, 3D EPI

## Abstract

This review article examines the current state of BOLD fMRI at a high magnetic field strength of 7 Tesla. The following aspects are covered: a short description of the BOLD contrast, spatial and temporal resolution, BOLD sensitivity, localization and spatial specificity, technical challenges as well as an outlook on future developments are given. It is shown that the main technical challenges of performing BOLD fMRI at high magnetic field strengths—namely development of array coils, imaging sequences and parallel imaging reconstruction—have been solved successfully. The combination of these developments has lead to the availability of high-resolution BOLD fMRI protocols that are able to cover the whole brain with a repetition time (TR) shorter than 3 s. The structural information available from these high-resolution fMRI images itself is already very detailed, which helps to co-localize structure and function. Potential future applications include whole-brain connectivity analysis on a laminar resolution and single subject examinations.

## 1. Introduction

Historically, MRI of the human brain has profited considerably from the transition towards higher field strengths. The benefits of the increased signal-to-noise-ratio (SNR)—and thus ability to acquire images at higher spatial resolutions and/or with reduced measurement time—have proven greater than the technical challenges such as the increased main field (B_0_) inhomogeneity [1,2]: Over the last decade, 3 Tesla systems have become readily available and now find use in many routine clinical applications. This paper is primarily concerned with field strengths of 7 T and higher that have attracted rapidly growing interest during the recent years. Among the neuroimaging applications, fMRI was expected to profit in more ways than just by an increase SNR. The blood oxygenation level dependent (BOLD) effect is based on microscopic magnetic field inhomogeneities in and around blood vessels that contain deoxyhemoglobin, and increases super-linearly with field strength [3]. Furthermore, the BOLD contrast should originate more strongly from the microvasculature, thus in summary, BOLD fMRI at high field should allow higher spatial resolution, be more sensitive and also more specific compared to lower field strengths. However, in order to reap these benefits of high field for fMRI, several technical hurdles had to be overcome first. This review article is structured such that first a short summary on the BOLD effect is given, and then the field strength related issues are discussed in separate sections.

## 2. The BOLD Effect

The observation that increased deoxyhemoglobin concentration leads to an increased contrast of blood vessels was first made in a rat brain and described by Ogawa *et al.* [4] in 1990. In a follow up study [5] the acronym BOLD was coined for this effect. Very soon afterwards, the first BOLD studies were used to perform mapping of brain function in humans [6,7] and firmly established the method as non-invasive alternative to the then commonplace use of positron emission tomography (PET). This triggered thousands of non-invasive functional imaging studies. In the initial period the fast low-angle shot (FLASH) sequence was used which yields good T_2_* weighted BOLD contrast, but in practice was restricted to single slice experiments because of the slow acquisition speed. With the implementation of stable gradient-echo echo-planar imaging (EPI) [8] an entire slice could be imaged with a single excitation, now allowing the whole brain to be covered with sufficient spatial (3–4 mm isotropic) and temporal (2–3 s) resolution.

**Figure 1:**
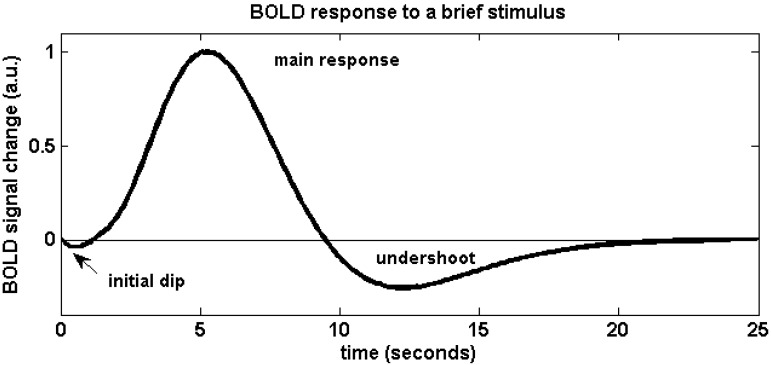
Schematic of the BOLD hemodynamic response to a brief stimulus at time zero. After the elusive “initial dip” that may arise as a result of initial oxygen uptake before hemodynamic changes occur, the blood flow effect dominates and causes the positive main BOLD response to peak after approximately 4–5 s. The return to baseline is typically preceded by a post-stimulus undershoot which can be of considerable duration.

**Figure 2:**
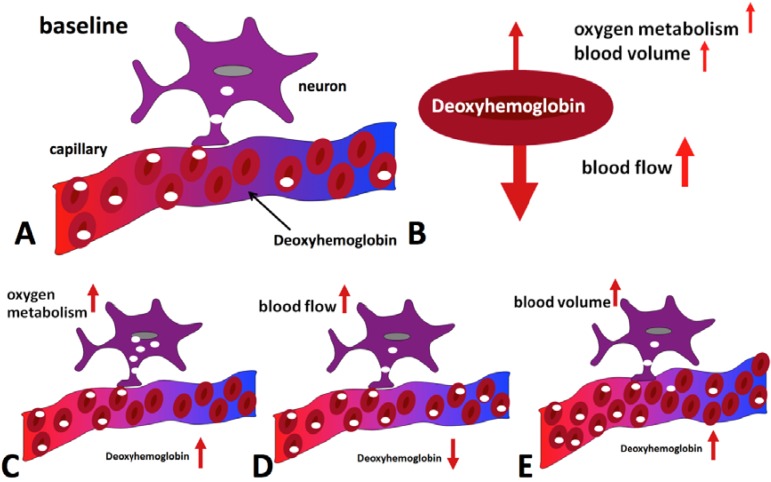
Hemodynamic effects contributing to the BOLD signal during activation. Panel (**A**) shows the situation in the baseline state, with only some of the delivered oxygen being utilized. The diagrams at the bottom illustrate the effect of oxygen metabolism (**C**), blood flow (**D**) and blood volume (**E**) increases on deoxyhemoglobin concentration. Blood flow increase dominates, leading to a net reduction of deoxyhemoglobin (**B**) and an increase in MR signal is hence observed.

The physiological effects that lead to a change of blood oxygenation, and thus a BOLD signal change, can be briefly summarized as follows (for a more detailed review, see e.g., [9]): Active brain cells metabolize oxygen extracted from oxyhemoglobin that is contained in red blood cells delivered by the local capillaries. Because hemoglobin is diamagnetic when oxygenated but strongly paramagnetic when deoxygenated, the interplay of physiological (hemodynamic) processes results in a net change of the magnetic susceptibility of blood: a higher deoxyhemoglobin content leads to a distortion in the local magnetic field in and around the vessels and hence attenuates the MR signal intensity. Importantly, the local increase in oxygen consumption during neuronal activation triggers a strong and over-compensating increase in local cerebral blood flow (CBF), delayed by approximately 1–2 s, but also leads to changes of blood volume (CBV). Some studies have reported the elusive “initial dip” which is ascribed to the initial oxygen uptake before hemodynamic changes set in; however the effect is disputed. The combined interplay of oxygen extraction, CBV and CBF results in a hemodynamic response function (HRF) that reaches a peak after 4–5 s. After stimulus cessation the so-called post-stimulus undershoot is typically observed before the signal returns to baseline value. This undershoot is maximal approximately 12–15 s post stimulus. Its duration depends on the duration and intensity of the preceding neuronal activation, as well as the brain region involved, and has been reported to last up to 40 s for intense visual stimuli. During the main response, the change in CBF is the by far dominant effect and serves to increase blood oxygenation by effectively “washing out” the deoxyhemoglobin produced during activation. A BOLD signal *increase* is therefore observed. A schematic of the BOLD response can be found in Figure 1, which was generated using FMRISTAT software [10] with the HRF impulse response parameters proposed by Glover *et al.* [11] and by addition of an initial dip. Figure 2 illustrates the different hemodynamic changes that take place during neuronal activation. 

Changes in CBF and CBV can also be measured directly and non-invasively by MR using specifically designed pulse sequences based on arterial spin labeling [12,13] and vascular space occupancy [14], respectively, but their generally lower sensitivity than BOLD renders them less suitable for most routine fMRI applications.

## 3. Spatial and Temporal Resolution and BOLD Sensitivity

Naturally, it is desirable to achieve as high a spatial resolution as possible, so as to potentially better localize functional activation. There are, however, several issues that have to be considered. The signal from a voxel is inversely proportional to its volume, *i.e.*, (voxel length)^3^; this means for example that an increase in resolution by a factor of two along all three dimensions leads to a signal decrease by a factor of eight. As signal-to-noise ratio (SNR) increases roughly linearly with field strength, scanning at higher field strength allows obtaining higher spatial resolution without compromising sensitivity. On the other hand, high resolution EPI requires a larger echo train length (ETL), and the absolute B_0_ inhomogeneity increases linearly with field strength. As a consequence, geometrical distortions in EPI images get considerably worse as the distortion is proportional to both ΔB_0_ and ETL. This can complicate the coregistration of functional and anatomical images. Last but not least, increasing resolution increases the amount of data to be sampled and, consequently, acquisition time. 

One might argue that temporal resolution is of less importance as the BOLD HRF is slow and acts as a low pass filter of the signal, so that a volume repetition time (TR) of 2–3 s is sufficient for almost all fMRI applications. Despite the slow BOLD HRF certain neuroscientific questions such as temporal delays can be investigated by using short TR, see e.g., [15,16]. However, when high resolution is desired with standard EPI, the increasing number of slices needed for whole-brain coverage can make it difficult to achieve a volume TR of 3 s. There are two additional arguments why a higher temporal resolution should be beneficial. First, nuisance signals from breathing and heartbeat are more completely sampled and can therefore be corrected for more effectively in a post-processing step. Correction can be achieved by fitting to the separately acquired respiration and cardiac signals in k-space or image space, for instance using RETROICOR (retrospective image correction) [17], or by including the physiological signals in the statistical model during subsequent analysis. Second, more sampling points increase the statistical power in the analysis, and a sequence with shorter $TR_{volume}$ is therefore desirable [18]. In the limit of Gaussian distributed temporal noise the power gain is proportional to $\sqrt{N}\propto 1/\sqrt{TR_{volume}}$, with somewhat lower gains expected in practice due to the temporal auto-correlation of physiological effects. Typically, increased temporal resolution requires a compromise on spatial resolution when keeping other parameters the same.

The parameter that one is interested in maximizing for BOLD fMRI is BOLD sensitivity (BS). In general, BS is determined by the BOLD signal change between activation and rest (“contrast”) [19]. Here we define an integral BS that also includes temporal noise and temporal resolution to reflect all parameters that influence statistical analysis: $BS=tCNR/\sqrt{TR_{volume}}$. This comparative measure is approximate in the presence of non-Gaussian temporal noise. In case one keeps *TR_volume_* fixed (as we will do in the following section), temporal contrast-to-noise ratio (tCNR) can be used as the parameter of interest. 

## 4. Estimation of the Dependence of BOLD Sensitivity on Field Strength

In the following we want to perform a more detailed estimate of the field strength dependence of tCNR, for both gradient and spin echo contrast. For the sake of simplicity we will use R2 (1/T2) as relaxation rate in all equations; for gradient echoes one only has to replace R2 by R2*. To estimate tCNR or tSNR, one has to also estimate temporal noise ($\sigma_{t}$), which contains both thermal noise ($\sigma_{0}$) and physiological fluctuations ($\sigma_{p}$) that are caused by breathing, cardiac and motion related influences. It has been shown that physiological effects are far more prominent than thermal noise especially when using large voxel volumes and high field [20]. It is thus very important to find solutions to minimize physiological “noise”. One very effective way to achieve this is to increase resolution and thereby shift the dominant noise contribution in a voxel from the physiological to thermal regime [20,21]. By spatially smoothing one can regain some temporal SNR lost by acquiring smaller voxels [22]. However, the maximum achievable temporal SNR (tSNR) per voxel is limited by image SNR (SNR_0_).

tCNR is equal to $\Delta S/\sigma_{t}$ with $\sigma_{t}=\sqrt{{\sigma_{0}}^{2}+{\sigma_{p}}^{2}}$; ΔS is the signal change between an activated state and a baseline or reference. In the simplest case the MR signal can be written as $S=S_{0}\cdot e^{-TE\cdot R_{2}}$. Assuming small changes in R2, ΔS can be approximated as $\Delta S\approx S_{0}\cdot TE\cdot \Delta R_{2}\cdot e^{-TE\cdot R_{2}}$. Using $\sigma_{p}=\lambda \cdot S$ and SNR_0_ = S/σ_0_, tCNR is given by

(1) $$tCNR\;\approx \frac{\text{TE}\cdot \Delta R_{2}}{\sqrt{1/SNR_{0}^{2}+\lambda^{2}}}$$

λ can be assumed—and has also been shown—to be B_0_ independent [20], SNR_0_ increases linearly with B_0_ and TE is normally chosen to match the tissue (gray matter) T_2_(*). Thus, the field dependence of the BOLD sensitivity should solely be reflected by ΔR_2_(*), which, however, strongly depends on tissue type (large veins, microvasculature). Theoretically the dependence of ΔR_2_(*) on B_0_ should be linear for larger veins and quadratically for microvasculature [23]. More recently, it has been shown that the expansion of the field dependence of R_2_ can even include terms of B_0_ to the power of five [24]. Experimental evidence is less clear, and studies have shown both supra-linear [25] and linear increase of ΔR_2_(*) for larger veins and linear and super-linear increase for gray matter [3,26,27]. Most probably, these inconsistent findings are due to different tissue types present in a voxel and thus spatial resolution. The spatial resolution currently used in functional imaging studies as well as subsequent smoothing leads to a mix of contributions which might explain some of the discrepancy between the (at least) linear increase in tCNR predicted by Equation 1 and the supra-linear gain in BS that has been found (see e.g., References [20,27,28]). In addition, the estimation in Equation (1) also does not take into account differences in experimental setup (e.g., instrumentation, coil design) at different field strengths and neglects changes in blood volume during activation. 

## 5. Localization and Spatial Specificity

This section contains a description of how the different (primarily venous) vessel structures impact on the spatial localization and functional specificity in BOLD fMRI. The BOLD signal is mainly composed of signal contributions from larger veins, smaller venules, and capillaries, *i.e.*, where deoxyhemoglobin concentration changes significantly. 

It has been shown that larger venous vessels give rise to a significant portion of the observed BOLD activation both in gradient-echo fMRI [29,30,31,32,33,34,35] and also spin-echo fMRI [36]. This leads to spurious “activation” and an intrinsic degradation of the functional localization and spatial specificity, as significant signal changes may occur far from the site of neuronal activation. Increased spatial specificity in BOLD fMRI thus cannot be achieved by simply increasing nominal spatial resolution. However, as demonstrated in the previous section, the size of the BOLD signal originating from the capillaries increases roughly as the square of the magnetic field strength compared to an “only” linear increase for larger veins. This is confirmed by experimental results indicating that the BOLD signal can be weighted towards the smaller vessels, and should hence be closer to the neuronal events, by using higher magnetic fields [3].

As the venous part of the vasculature is relevant for BOLD fMRI, knowledge about venous vasculature is very important and can be summarized as follows: Venous blood from the capillaries is first drained by venules (10 μm diameter) within a vascular layer within cortical gray matter (four such vascular layers have been defined by [37]) to an intracortical vein (diameter ~80 μm) that connects the vascular layers—runs hence perpendicular to the venules—and leads to the cortical surface. Here these intracortical veins emerge from the cortex roughly at a right angle and connect to the extracortical pial vein network (vein diameter >200 μm). The venous blood thus drains along the cortical surface, leading away from the site of neuronal activation. From this anatomical argument it becomes clear that functional signal changes detected in large extracortical veins can severely compromise functional localization and specificity. The estimates for the distance at which “functional activation” can still be detected range from 4.2 mm [38] to three times that amount [39] when including more recent findings on vascular properties of human gray matter microcirculation into the earlier model. Boxerman *et al.* [40] showed by using Monte-Carlo simulations with a cylindrical vein model that a spin echo sequence is primarily susceptible to capillaries whereas a gradient echo sequence is most sensitive to veins larger than capillaries (diameter >10 μm). 

The specific venous intracortial architecture explains why a layer specific activation can be found in GE BOLD fMRI (laminar fMRI). Once the resolution is sufficiently high one can either remove the extracortical vein contributions from the activation based on the low venous signal intensity [29] or one can perform laminar (cortical profile) analysis, so that one can eliminate extracortical veins and their contribution on position alone [41,42,43]. Another possibility is to reduce the contribution of large veins by using SE based sequences, which are mainly sensitive to the extravascular contributions around the microvasculature. Also the intravascular T_2_ contribution is reduced due to the short T_2_ of venous blood at higher fields. However, the overall SE BOLD sensitivity in cognitive paradigms has been reported to reduce by about a factor of three with respect to a comparable GE sequence at 3 Tesla [44,45]. Nevertheless, it has been possible to map cortical columns at 7 T by using SE-EPI [46] thanks to its better spatial specificity and increased relative contribution of T_2_ contrast at high field. For a more detailed discussion on the differences of SE and GE contrasts see [9]. 

## 6. Technical Challenges

Some of the technical challenges researchers were facing when first using high magnetic field strengths (≥7 Tesla) for *in vivo* MRI were similar to those previously encountered when the stepping to a higher field strength, *i.e.*, larger B_0_ and B_1_ inhomogeneity and quadratically increased RF power. While main and transmit field inhomogeneity remained manageable for the brain at 3 Tesla, they became severe at 7 Tesla causing severe degradation of image quality. 

The problem of B_0_ inhomogeneity can be addressed by either by performing a few (additional) repetitions of a conventional 3D B_0_ map shim [47], or by using an automated shim procedure that is restricted to the volume of interest [48]. However, even with a good shim local B_0_ inhomogeneity e.g., in the prefrontal brain remains much greater than at lower fields. In T_2_* weighted fMRI inhomogeneity perpendicular to the slice causes signal dephasing that leads to irreversible signal loss. An effective way to reduce the problem is the use of thinner slices—with the added benefits of increased resolution. For instance, with dephasing along the slice direction, reduction of the slice thickness at 7 Tesla by about a factor of two as compared to 3 Tesla, the signal dropout will be similar. Of course, the increased number of required slices will lead to an increased measurement time or decreased volume coverage if no other measures are taken. Also increasing the in-plane spatial resolution is demanding as with increasing field strength one faces the need to reduce the echo time in order to match the shorter T_2_*. The shorter TE puts a lower limit on the achievable echo train length and hence matrix size and spatial resolution. Further increases in resolution are then only possible by reducing the amount of k-space that is acquired, e.g., by using partial Fourier imaging, partial parallel imaging, segmented acquisitions, or a combination thereof. Parallel imaging methods such as sensitivity encoding (SENSE) [49] or generalized auto-calibrating partially parallel acquisitions (GRAPPA) [50] proved to offer a very efficient way of dealing with the problem once suitable coil arrays had become available. Using acceleration factors of 2–4 is now possible with very acceptable image quality, and thanks to the temporal stability with only negligible effects on functional sensitivity [51,52].

Counterintuitively, instead of the expected reduced measurement times due to shorter T_2_* and higher signal, a prolongation is often necessary as a consequence of the increase in RF power deposition that pushes even common GE EPI protocols beyond SAR limits; much of the RF power goes into fat saturation pulses to remove the fat signal in the images. One way to circumvent the restriction is to use longer TR and/or to limit spatial coverage to the brain region of interest. A more practical solution is to use lower flip angles for fat saturation which has shown to be sufficient [53] or even to disable fat suppression entirely [54] which is often acceptable thanks to the short T_2_* of fat at high field. 

B_1_ (transmit field) inhomogeneity increases with increasing field strength as the RF wavelength becomes short in comparison to the typical dimension of the object (e.g., head) to imaged. This leads to an inhomogeneous B_1_ field, and hence flip angle and intensity distribution that is sometimes referred to as “central brightening” [55]. B_1_ inhomogeneity was historically expected to become a fundamental limitation of high-field MRI, in practice however it is not a severe problem for current applications GE BOLD fMRI. The central hyper-intensity may even be regarded as an advantage as it is roughly complementary to the receiver sensitivity variation of currently typically used coil arrays, thereby aiding a more homogenous SNR distribution. By contrast, applications based on spin echoes suffer considerably, as perfect spin refocusing is not achieved and the precise contrast hence not known. Several techniques for “B_1_ shimming” now begin to be used routinely, including special RF pulse designs and multi-channel transmission of amplitude and phase modulated waveforms [56,57,58,59].

Increased spatial coverage (in particular, number of acquired slices) inevitably leads to a proportional increase in TR and total measurement time if the same number of scans is to be acquired. Only recently strategies have been proposed that address this issue. The acquisition time for many slices in 2D EPI can be reduced by using “multiplexed” EPI (M-EPI) acquisitions whereby multiple slices are excited and sampled simultaneously, such as the simultaneous echo refocusing (SIR) method [60], multiband excitation [61,62] and their combination [63]. Simultaneous multi-slice excitation results in multiple slices being aliased into a single image, which can subsequently be disentangled using analogues of SENSE or GRAPPA parallel reconstructions [64]. Whole brain coverage at typical spatial resolution has recently been achieved in less than about 0.4 s, great improving the statistical power of fMRI analyses [63]. RF power deposition remains a potential limitation as SAR per slice is approximately the same and hence total SAR increases with decreasing TR. The novel multi-slice technique PINS (power independent of number of slices) alleviates the SAR constraint by the use of periodic rather than distinct limited multi-band excitation at high field [65].

**Figure 3:**
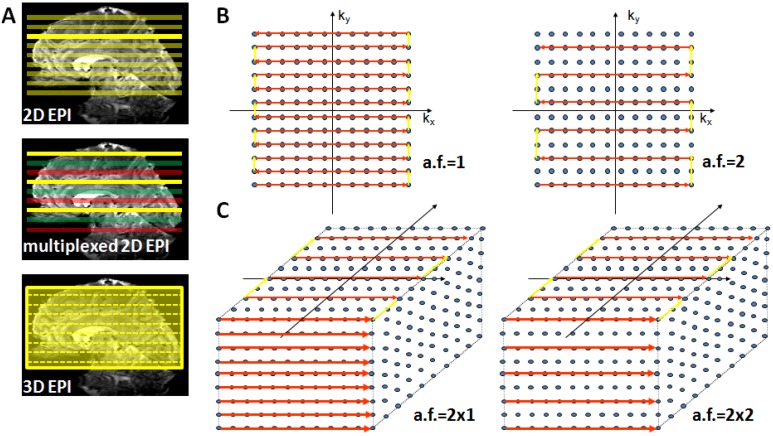
Suitable echo-planar imaging (EPI) sampling schemes for high-field fMRI. Panel (**A**) top: Conventional 2D EPI where every slice is excited and acquired separately; middle: in multiplexed EPI several slices are excited simultaneously (colors indicate slice groups), allowing an acquisition speed-up given by the multiplexing factor; bottom: in 3D EPI the slice direction is replaced by a secondary phase encoding direction and the entire volume is continuously excited. With full sampling the speed is identical to 2D EPI, but parallel undersampling along k_z_ is now possible and allows substantial repetition time (TR) reductions. Panel (**B**) illustrates full (left) and factor-two accelerated (right) in-plane k-space sampling with 2D EPI. Panel (**C**) shows 3D EPI sampling schemes with factor 2 × 1 (left) and factor 2 along both primary (k_y_) and secondary (k_z_) phase encoding direction (right).

Alternatively, the increasingly longer acquisition time of 2D EPI can be resolved by using a 3D GE-EPI method, which also enables parallel acceleration in the second phase encoding direction. This considerably shortens measurement time as current coil arrays (e.g., 24 or 32 channel head coils) permit acceleration factors of 2–3 in the second PE encoding direction where a time saving equal to the nominal acceleration factor is achieved [66]. Figure 3 provides a schematic illustration of conventional 2D EPI, multiplexed 2D EPI and 3D EPI and their implications for accelerated acquisitions. 

**Figure 4:**
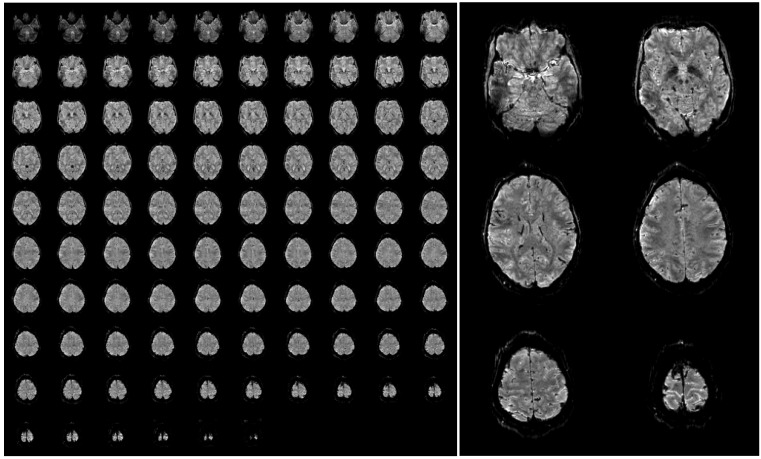
Example of a single 3D-EPI volume with 96 slices acquired in 2.3 s at a nominal resolution of 1 mm (**A**). A few slices have been selected and enlarged to be able to appreciate the fine anatomical details that are visible in a single functional image (**B**). The images have been bias field corrected using FMRIB’s Automated Segmentation Tool (FAST, FSL, [72] for visualization purposes. The complete acquisition parameters are: TR/TE/FA = 45 ms/17/ms/15deg, TR_vol_ = 2.34 s, matrix size 200 × 200, 96 slices + 9% slice oversampling, GRAPPA acceleration 4 × 2.

Furthermore, SAR is not an issue in 3D EPI as due to the short TR the flip angle is very small (only around 15 degrees assuming the Ernst angle for optimal gray matter signal). A further reduction in SAR and measurement time can be obtained by replacing the standard fat suppression by binomial pulses that make use of the dephasing between water and fat. These measures enable whole brain coverage with an isotropic resolution of 1 mm in less than 2.5 s. An example of such high-resolution 3D EPI data acquired at 7 Tesla is shown in Figure 4, to illustrate the achievable image quality and the strong inherent anatomical contrast.

The disadvantage of a 3D acquisition is that the available magnetization is reduced due to the short TR and that it is essentially a multi-shot method. The SNR of 3D acquisitions increases with $\sqrt{N_{slices}}$ and it has been shown at 7 T that the reduced magnetization is therefore more than compensated when a large volume (*i.e.*, a large amount of slices) is acquired. The discussion of the issues of multi-shot acquisitions is beyond the scope of this paper and we refer to [67,68,69,70,71]; however, experience has shown that acquisition of k_z_-planes in a single shot (*i.e.*, segmentation only along the second phase encoding—the slice—direction) appears to be very advantageous regarding the reduction of ghosting artifacts. 

One main unresolved issue at 7 Tesla and higher is the application of whole brain SE-EPI. Up to now various studies have demonstrated increased spatial specificity by using high spatial resolution in a restricted region of the brain; it would be advantageous to be able to employ this high resolution in whole brain studies, and without the severe SAR restrictions currently inherent to the use of SE.

## 7. Summary and Outlook

To summarize, it was shown that the main technical challenges of performing BOLD fMRI at high magnetic field strengths (7 Tesla) have been solved successfully during the last decade. The main contributing factors were the development of array coils, both on the receiver (coil arrays with many receive elements) as well as on the transmit side, the adaptation of imaging sequences, and robust parallel imaging implementations. These developments are especially useful for the implementation of robust protocols when going to even higher field strengths (9.4 Tesla and above). 

The combination of these developments has lead to the availability of high resolution BOLD fMRI protocols that are able to cover the whole brain with a TR that allows for event related stimulus paradigms, *i.e.*, a volume TR shorter than 3 s. The structural information available from these high-resolution fMRI images itself is already very detailed. This is particularly promising as one can therefore acquire high-resolution anatomical and structural connectivity information in the same scan, which, moreover, alleviates the problem of distortion when overlaying functional and structural data. Future applications may include whole-brain connectivity analysis using DCM (dynamic causal modeling) on a spatial scale in the order of the cortical laminae and applications that demand sufficient sensitivity on a single subject level.
